# Anaerobic Co-Digestion of Pig Manure and Rice Straw: Optimization of Process Parameters for Enhancing Biogas Production and System Stability

**DOI:** 10.3390/ijerph20010804

**Published:** 2023-01-01

**Authors:** Pengjiao Tian, Binbin Gong, Kaijian Bi, Yuxin Liu, Jing Ma, Xiqing Wang, Zhangsun Ouyang, Xian Cui

**Affiliations:** 1 College of Food Science and Technology, Hubei University of Arts and Science, Xiangyang 441053, China; 2 College of Life Science, Xingtai University, Xingtai 054001, China; 3 Jiangxi Rural Energy and Enviroment Agency, Nanchang 330031, China; 4 State Key Laboratory of Food Science and Technology, Engineering Research Center for Biomass Conversion, Ministry of Education, Nanchang University, Nanchang 330047, China

**Keywords:** Anaerobic co-digestion, rice straw, pig manure, biogas, model analysis

## Abstract

The objective of this study was to optimize the process parameters of the anaerobic co-digestion of pig manure and rice straw to maximize methane production and system stability. In this study, batch experiments were conducted with different mixing ratios of pig manure and rice straw (1:0, 1:1, 1:5, 1:10, and 0:1), total solid concentrations (6%, 8%, 10%, 12%, and 14%), and inoculum accounts (5%, 10%, 15%, 20%, and 25%). The results show that a 1:5 mixing ratio of pig manure to rice straw, a 12% total solid content, and a 15% inoculum account yielded biogas up to 553.79 mL/g VS, which was a result of co-digestion increasing the cooperative index (CPI > 1). Likewise, the evolution of the pH and VFAs indicated that the co-digestion system was well-buffered and not easily inhibited by acidification or ammonia nitrogen. Moreover, the results of the Gompertz model’s fitting showed that the cumulative methane production, delay period, effective methane production time, and methane production rate under optimal conditions were significantly superior compared to the other groups employed.

## 1. Introduction

With the rapid development of intensive farming, vast quantities of livestock and poultry waste have been generated, which now constitute the main source of agricultural, non-point source pollution [1]. Especially in areas with high concentrations of livestock and poultry, livestock and poultry waste has become a major source of environmental pollution [2]. In the United States, the waste produced by livestock and poultry farms is more than 130 times that of humans, which seriously threatens the local ecological environment [3]. In China, the stockpile of livestock and poultry manure is large, and its environmental impact is extensive; however, the utilization rate of livestock and poultry manure is low [4]. In some parts of China, the pollution of livestock and poultry manure and urine not only threatens the health of residents living in the urban-rural areas, but also leads to a large loss of nitrogen and phosphorus from farmland [4,5]. Therefore, it is necessary to develop an effective disposal strategy for livestock manure.

The anaerobic digestion (AD) of livestock manure is a potential resource treatment strategy [6]. During the AD process, organic matter is converted into renewable energy (biogas) and organic fertilizer under the action of microbial communities, thereby contributing to the utilization rate of the waste matter [7,8]. The AD technology using livestock manure as raw material has developed rapidly. Previous studies showed that the biogas production rate of the mesophilic AD process of livestock manure can reach 5 m^3^/(m^3^·d). However, livestock manure is characterized by higher N content, low C content, and a low C/N ratio, limiting its ability as a resource for biogas production [9,10,11]. Recently, anaerobic co-digestion (AcoD) was considered to be a promising solution for improving the performance of the single AD process [12,13,14]. Generally, AcoD provides a better macro- and micro-nutrient balance, better pH buffering capacity, superior dilution of toxic elements (heavy metals, pharmaceuticals, etc.), and proper moisture balance [14]. Likewise, AcoD contributes to the growth of a diverse microbial community in the digester and better process efficiency, thus leading to higher biogas production and stabilized digestate [15]. To date, various studies have investigated AcoD performance with different substrates. For instances, Morales-Polo et al. (2023) confirmed that the AcoD of different types of food waste can increase methane production and buffer acidification inhibition [13]. Likewise, Jiang et al. (2022) indicated that the biogas production rate via AcoD of food waste (fruit and vegetable wastes) and sewage sludge was higher than that in the mono-AD process [14]. Among the various substances used in the AcoD process, rice straw, as a typical lignocellulosic biomass, is considered to be one of the promising materials for the livestock manure AD process [16]. Rice straw contains higher C content and can balance the C/N ratio in the digestate-treated livestock manure [17]. Thus, the co-digestion of livestock manure and rice straw can prove to be beneficial with respect to achieving biogas production and stable process conditions. However, to date, the optimal process for the AcoD of livestock manure and rice straw is not clear.

To fill these knowledge gaps, pig manure and rice straw were selected as the substrates for co-digestion in this study. Likewise, the process parameters of AcoD, including different mixing ratios, substrate concentrations, and inoculum amounts, were optimized. The main objective of this study is to optimize the variable process parameters to enhance biogas production and process stability. Moreover, the experimental observations of the present study are validated with the Gompertz model.

## 2. Materials and Methods

### 2.1. Feedstock and Inoculum

The pig manure used in this study was obtained from a large-scale-pig-breeding company in Pingxiang, Jiangxi province. The waste was collected and stored in a freezer at 4 ℃. Rice straw was taken from the suburbs of Nanchang City, Jiangxi province, after being harvested and naturally dried. Then, the rice straw was crushed to a length of 2–3 cm with a 9Z-2.5 crusher (Zong Vision Technology Co., Ltd., Beijing, China) and stored for use. Moreover, the inoculum was taken from a long-running biogas plant in Pingxiang City. The characteristics of the pig manure, rice straw, and inoculum are listed in Table 1.

### 2.2. Setup of the Anaerobic Digestion Experiments

**Figure 1:**
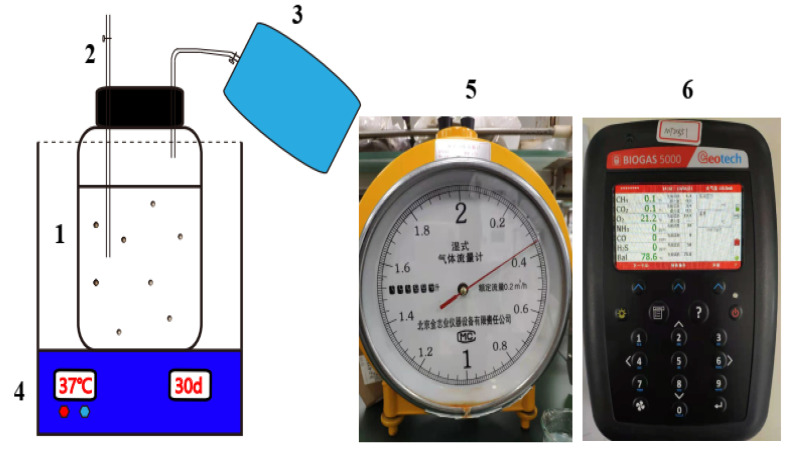
The batch anaerobic digestion reactor. (1. Anaerobic digestion reactor; 2. Sampling port; 3. Methane collection bag; 4. Water bath; 5. Gas flow meter; 6. Methane analyzer).

In this study, the co-anaerobic digestion experiment was conducted according to VDI standard procedure 4630 [18]. Each treatment was replicated three times and the samples were fermented for 30 days in a 1 L mesophilic anaerobic digestion reactor. The reactor is mainly composed of anaerobic digestion reactor, biogas collection device, and temperature control device (Figure 1). The effects of mixing ratio (1:0, 1:1, 1:5, 1:10, and 0:1), TS concentration (6%, 8%, 10%, 12%, and 14%), and inoculum amounts (5%, 10%, 15%, 20%, and 25%) on the co-digestion performance were investigated. Experiments with different mixing ratios were first carried out, using the 8% substrate concentration and 15% inoculum amount. Then, based on the optimum mixing ratio, experiments with different substrate concentrations were carried out with an inoculum of 15%. Finally, the effect of inoculum amount on co-digestion was investigated based on the optimum mixing ratio and substrate concentration. The material and inoculated sludge were added to the reaction flasks according to the experimental design, and the total loading volume was adjusted to 800 g by adding water. After the fermentation material and liquid were mixed well, nitrogen was introduced to exclude air and then the flasks were sealed to ensure an anaerobic environment; then, parallel sampling groups were set up for each treatment. The fermentation unit was placed in a water bath at 37 ℃ ± 2 ℃ for anaerobic digestion at medium temperature, and the fermentation cycle was 30 days. During the anaerobic fermentation period, samples were taken on days 1, 3, 5, 7, 10, 15, 20, and 30, and the post-fermentation liquor was collected from parallel groups at each sampling point, centrifuged, filtered, and then tested for pH, VFAs, and other physicochemical indicators. The daily gas production and gas composition (CH_4_ and CO_2_) were measured at the same time every day, and the headspace correction of daily gas production was performed according to the German anaerobic digestion standard VDI 4630. The cumulative gas production was calculated at the end of anaerobic digestion, and kinetic fitting was performed to analyze the stability and gas production characteristics of the anaerobic digestion process under different operating conditions. Manual shaking and stirring was performed at 9 am and 9 pm every day during the experiment. The gas collection bags were taken down and replaced at the same time each day. Daily biogas production was measured with a wet gas flow meter and the methane content was measured with a biogas tester.

### 2.3. Analysis Methods

The pH value was measured via a standard pH meter (PHS-2F, OUSTOR, Shanghai). For TS, the samples were kept in the oven and dried at 105 °C until attaining a constant weight, and for VS, the oven-dried samples were kept in a muffle furnace for ignition at 550 °C. For sCOD, the supernatant was passed through glass filter paper with a 0.45 μm pore size. TOC was measured by the Walkley–Black Method [19] using the diphenylamine indicator and titrating against the 0.5 N-Mohr salt solution. The total carbon (TC) and total nitrogen (TN) content of the samples was determined by the elemental analyzer (Multi N/C 3100, Germany). NH^4^-N was analyzed by lixiviating the dry sample with 2M-KCl and following the phenate method as per standard methods. Then, content of cellulose, hemicellulose, and lignin was determined by Normal form wasting method. Briefly, 0.5 g dry sample was taken and placed into a mesh bag (F57, ANTOM, USA); then, it was examined via ANTOM 220 cellulose analyzer (Beijing, China). Volatile fatty acids were determined by high-performance liquid chromatography (HPLC-1260, Agilent, Santa Clara, CA, USA) for acetic acid, propionic acid, and butyric acid [20]. Standard acids were prepared as standard curves for quantitative analysis. Before determination, the samples were subjected to the following pretreatment: the anaerobic fermentation broth was centrifuged and filtered, the supernatant was mixed 1:1 with chromatographically pure ethyl cyanide, left for 5 min to allow for complete precipitation of the proteins, and the supernatant was centrifuged at 12,000 rpm for 30 min and injected into a liquid phase vial over a 0.22 μm organic system filter membrane to be measured. For determination, the injection volume was 10 μL, and the mobile phase was 0.05 M sulfuric acid at a flow rate of 0.6 mL/min. Separation was performed on an HPX-87 (300 × 7.8 mm, Bio-Rad, Hercules, CA, USA) column with a column temperature and injection temperature of 30 ℃ and a column pressure of 6.3–6.8 Mpa. VWD) at 210 nm to generate a liquid phase spectrum. The peak times of acetic acid, propionic acid, and butyric acid were 4.15, 6.64, and 15.68 min in g/L, in that order.

### 2.4. Co-Anaerobic Digestion Synergy

Co-fermentation synergistic effect index (CPI) [21]: The methane production potential varied with the difference in organic nutrient composition of the materials with different mixing ratios. The actual degree of methane production of each experimental group was analyzed in comparison with the calculated values of the theoretical equation. The actual values of methane production from pig manure and rice straw fermentation alone in the experiment were determined to be 248.4 and 181.71 mL/g VS, respectively; then, the theoretical calculated values were weighted and superimposed according to the amount of methane production from different proportions of mixed substrates. The CPI can be calculated by the following Equation (1):

(1) $$CPI_{i,n}=\frac{B_{i.n}}{B_{oi,n}}=\frac{B_{i,n}}{\sum_{i}^{n}\%VS_{i}B_{oi,n}}$$

where *B_i,n_* is the methane production potential of mixed materials; *B_o,n_* is the methane production potential of a single material; and *B_oi,n_* is the weighted mean methanogenic potential of mixtures (%VS).

### 2.5. Kinetic Model Analysis

A modified Gompertz model was used to validate the degrees of methane production in this study. Briefly, the modified Gompertz model (Equation (2)) was used to fit the accumulative methane production data obtained from anaerobic co-digestion to predict the methane potential.

(2) $$M\left(t\right)=P⯀exp\{-exp[\frac{R_{m}⯀e}{P}⯀\left(\lambda -t\right)+1]\}\;$$

where *M* is the accumulative methane production (mL g^−1^ VS) at AD time *t* (d), *P* is the final accumulative methane production (mL g^−1^ VS), *Rm* is the maximum methane production rate (mL g^−1^ VS-d^−1^), *e* is Euler’s constant (2.7183), and *λ* is the lag phase time (days).

## 3. Results and Discussions

### 3.1. The Effect of Mixing Ratio on the AD Performance

**Figure 2:**
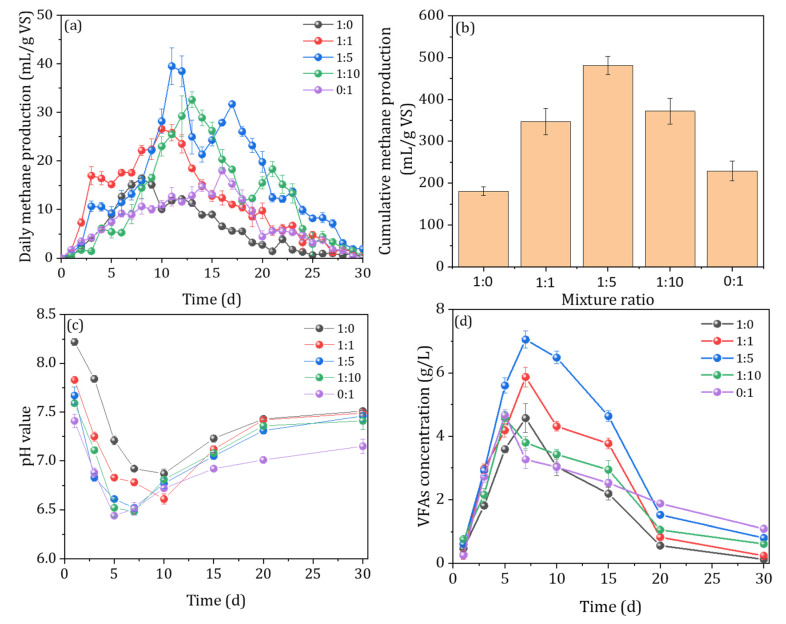
The daily methane production (**a**), cumulative methane production (**b**), the evolution of pH value (**c**), and VFA concentration (**d**) under different mixture ratios, 8% TS content, and 15% inoculum amount.

The effect of the pig manure and rice straw mixing ratios of 1:0, 1:1, 1:5, 1:10, and 0:1 on biogas production was investigated in this study. As shown in Figure 2a, a significant difference in daily methane production was observed among the different ratios of the pig manure and rice straw mixture. In detail, the maximum methane production increased first and then decreased with the increase in the rice straw proportion. The maximum daily methane production was observed at the mixing ratio of 1:5 (39.53 mL/g VS), which was 140.3, 48.6, 21.1, and 119.4% higher than the 1:0, 1:1, 1:10, and 0:1 mixing ratios, respectively. Moreover, a trend in which cumulative methane production increased and then decreased with the increase in the ratio of rice straw was observed (Figure 2b). The highest biogas production value (481.48 mL/g VS) was observed at a mixing ratio of 1:5, which was 166.7, 38.8, 29.5, and 110.3% higher than the 1:0, 1:1, 1:10, and 0:1 mixing ratios, respectively. The maximum cumulative methane production was determined at the ratio of 1:5, which further indicated that a suitable carbon to nitrogen ratio is beneficial for microbial activity and leads to a higher efficiency of substrate decomposition and utilization [22]. These results indicated the optimal ratio of pig manure to rice straw was 1:5 during the co-digestion process. Almomani et al. (2020) indicated that the AcoD of an agricultural solid waste and cow dung mixture resulted in a methane production value of 297.99 mL/g VS at mixing ratio of 3:2, which was higher than a 1:4 ratio [23].

Furthermore, the degrees of evolution of pH and VFAs were analyzed to further explore the stability of the co-digestion process (Figure 2c,d). The pH values of the different ratio groups decreased first and then increased gradually. The pH values decreased significantly during the first 7–10 days, which was mainly caused by the production of organic acids during the hydrolysis and acidification of organic substances at the early stage of the AcoD process [24]. However, the pH values gradually rebounded after 10 days and fluctuated within a stable range, which can be ascribed to the self-buffering ability of the AD system. Generally, as fermentation proceeds, the VFAs are gradually used and consumed by methanogenic bacteria, resulting in the pH of the system gradually rising back to equilibrium, which is also consistent with the previous studies [25]. During the whole fermentation cycle, the pH fluctuated within a stable range of 6.4–7.9 under all conditions, which was in the range of a suitable pH for anaerobic digestion, indicating that it was not overly acidic or overly alkaline, and the fermentation system operated normally without inhibition. Moreover, a significant difference regarding the VFA content was observed among the different ratios of the pig manure and rice straw mixture. The highest peak of the VFA content was observed at the mixing ratio of 1:5 (5997.61 mg/L), which was 67.1, 15.7, 47.5, and 125% higher than the 1:0, 1:1, 1:10, and 0:1 mixing ratios, respectively. This finding indicated that co-anaerobic digestion could improve the accumulation of VFAs, especially with respect to the content of acetic acids. Generally, acetic acid is the main precursor for methane production [26]. Thus, co-anaerobic digestion can significantly improve the stability of the system and promote the balance of VFAs production and consumption, especially with the efficient use of the methanogenic precursor substance acetic acid, thus enhancing the efficiency of methane production.

### 3.2. The Effect of TS on the AD Performance

**Figure 3:**
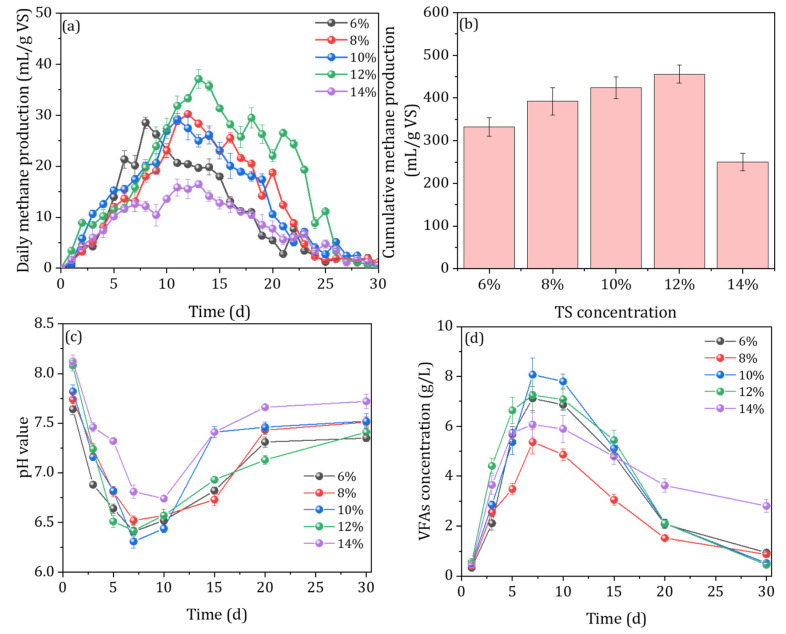
The daily methane production (**a**), cumulative methane production (**b**), the evolution of pH value (**c**), and VFA concentration (**d**) under different TS concentrations, 1:5 mixing ratio, and 15% inoculum amount.

The daily methane production and cumulative methane production at different initial substrate concentrations are shown in Figure 3. The results showed that the daily methane production was the highest when the substrate concentration was 12%, which was 30.02%, 22.88%, 26.95%, and 125.31% higher than the other groups. When the initial substrate concentration was 14%, the daily methane production was basically maintained at a lower level, and the peak daily methane production was the smallest. This suggests that higher substance concentrations limit the methanogenic fermentation process [6,27]. Likewise, a similar trend in the cumulative methane production was observed at the different substrate concentrations (Figure 3b). The results show that the cumulative methane production tends to increase and then decrease when increasing the initial substrate concentration. The highest cumulative methane production was observed at the substrate concentration of 12% (455.86 mL/g VS), which was 37.3, 16.3, 7.4, and 82.4% higher than the other groups, respectively. This may be due to the fact that high substrate concentrations limit mass transfer within the system, making it more difficult for dissolved organic matter to come into contact with microorganisms and reducing methanogenic efficiency. An et al. (2017) reported that biogas yield increased with a TS% increase from 2 to 8% and declined with a further increase in TS% [28]. Likewise, previous studies observed that the methane content in biogas decreased from 85 to 50% as the concentration increased from 10 to 50% (on VS basis) [29].

The changes in pH and the content of VFAs during AcoD with different initial substrate concentrations are shown in Figure 3c,d. During the AcoD process, the pH fluctuated within the normal range of 6.31–8.12 under all conditions, indicating that the fermentation system was operating normally (Figure 3c). Moreover, the highest peak in the VFA content was observed at the substrate concentration of 12% (5248.84 mg/L), which was 42.3, 39.3, 3.6, and 61.7% higher than the 6, 8, 10, and 14% TS concentrations, respectively. Likewise, the percentages of acetic acid were 62.60%, 60.10%, 77.80%, 74.86%, and 59.27%, respectively. These results indicated that the accumulation of VFAs and the conversion of acetic acid could be promoted by appropriately increasing the initial concentration of the substrate. When the substrate concentration was 12%, the concentration of acetic acid was greater than 1200 mg/L for an extended period, indicating that the microorganisms continuously degraded and transformed organic matter to produce acetic acid, constituting a process that plays an important role in the continuous production of methane [30]. As judged from the gas production and VFA indicators, the acidification and methanation processes of the system were no longer balanced when the substrate concentration was greater than 14%, indicating that the optimal initial substrate concentration was 12% when pig manure and rice straw were co-digested anaerobically at a ratio of 1:5.

### 3.3. The Effect of Inoculum on the AD Performance

**Figure 4:**
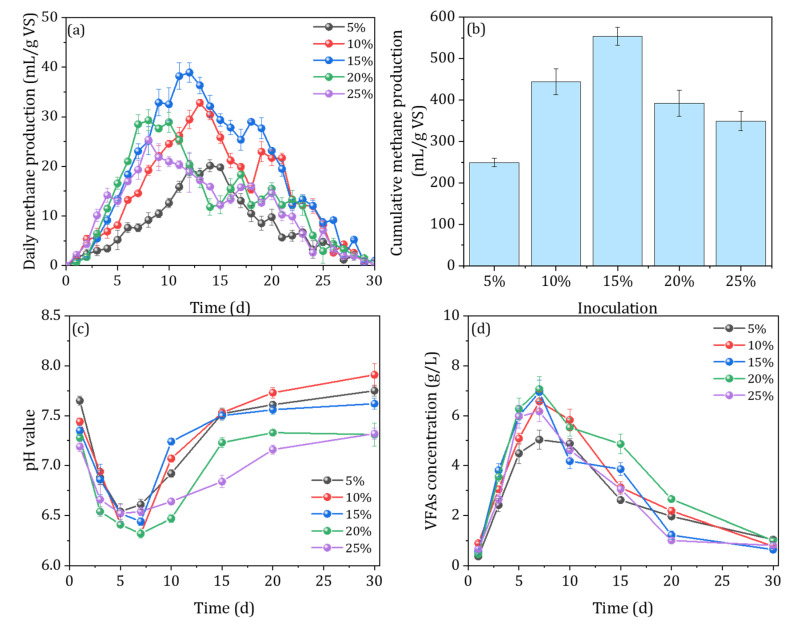
The daily methane production (**a**), cumulative methane production (**b**), the evolution of pH value (**c**), and VFA concentration (**d**) under different inoculation amounts, 1:5 mixing ratio, and 12% TS content.

The variation pattern of daily methane production with time at different inoculum levels is shown in Figure 4. The maximum daily methane production peaks were observed when the inoculum level was 15% (38.91 mL/g VS), which was 93.3, 18.7, 24.7, and 53.6% higher than at the 5%, 10%, 20%, and 25% inoculum levels, respectively. The results showed that with the increase in the amount of inoculum, the peak of daily methane production appeared earlier, and the maximum daily methane production showed a trend of increasing and then decreasing, which is consistent with the previous studies. Parra et al. (2021) explored the effect of different inoculum concentrations on the solid anaerobic digestion of asparagus. The results indicated that increasing the inoculum level could significantly shorten the delay period of anaerobic fermentation, which can be explained as the higher inoculation level having increased the abundance of bacteria community in the system, thus improving the efficiency of the hydrolysis, acidification, and methane production phases. However, at lower inoculum levels, methanogenic bacteria were less abundant and grew relatively slowly, which resulted in the relatively slow conversion of acetic acid, hydrogen, and carbon dioxide to methane by methanogenic bacteria [31]. As shown in Figure 4b, the cumulative methane production increased first and then decreased with the increase in the inoculation amount. When the inoculation amounts were 5%, 10%, 15%, 20%, and 25%, the cumulative methane production values were 248.95, 444.27, 553.79, 392.05, and 349.25 mL/g VS, respectively. The highest methane production was achieved at 15% inoculation, which was 1.22, 0.24, 0.41, and 0.58 times higher than that of the other groups, respectively. In general, increasing the inoculum amount within a certain range can both shorten the initiation time of the AD reactor and increase methane production. This is because the increase in the inoculum quantity accelerates the hydrolysis and acidification reaction rates of the anaerobic digestion substrate, which provides more sufficient raw materials for methanogenic bacteria. At the same time, increasing the inoculum amount can introduce more methanogenic bacteria and other microorganisms, thus increasing methane production [32].

The pH change patterns were similar among the different treatments, and the inoculum amount had no significant effect on the trend in the pH change in the AD system (Figure 4c). However, the larger the inoculum amount, the smaller the change in the pH of the system, indicating that the inoculum can regulate the pH of the AcoD system, as well as enhance the buffering capacity and provide a good environment for the microbiota [33]. Moreover, the inoculation ratio had no significant influence on the change trend of the VFAs (Figure 4d). The content of VFAs in each treatment reactor increased first and then decreased. The peak values, which appeared around the 10th to 15th day, were 3480.06, 5086.05, 6087.61, 4266.05, and 5066.14 mg/L, for which acetic acid accounted for 59.66%, 65.95%, 56.65%, 67.32%, and 66.17%, respectively. However, the concentration of VFAs in the system increased with the decrease in the amount of the inoculum, as increasing the amount of inoculum can introduce more microbiota, thus serving to consume VFAs in the system. At the same time, the relative content of acetic acid was smaller at lower inoculum levels, indicating that the inoculum level would influence the composition of VFAs, which is consistent with the previous studies [34].

### 3.4. Model Fitting Analysis of Anaerobic Digestion System

**Figure 5:**
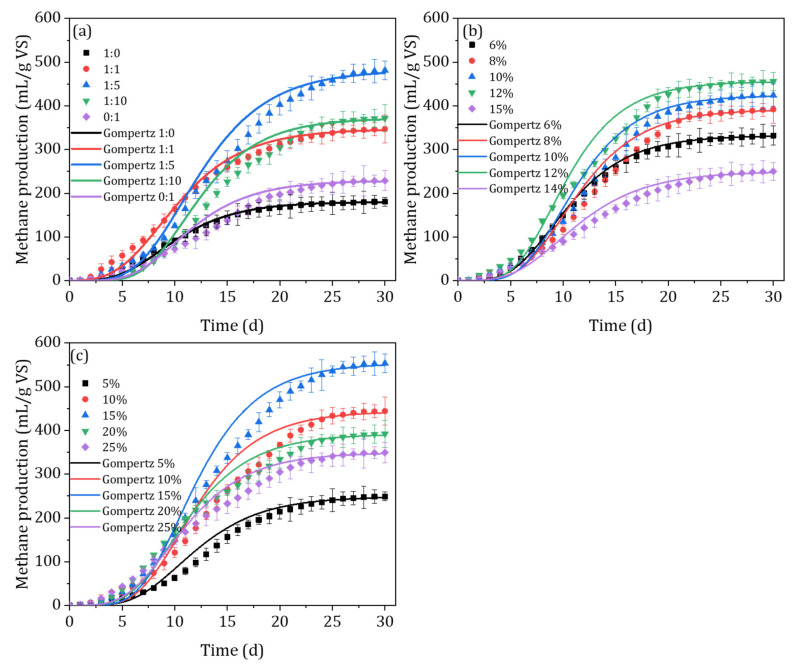
The results of Gompertz model analysis of the potential methane production under different mixing ratios (**a**), TS concentrations (**b**), and inoculum amounts (**c**).

In this study, the modified GM model was used to fit the AcoD process (Figure 5 and Table 2). The results showed that the R^2^ of the model fit index was greater than 98% for each mixing ratio of pig manure to rice straw, indicating that the modified GM model could accurately simulate the methanogenic process of the AcoD processes. When the mixing ratios of pig manure to rice straw were 1:0, 1:1, 1:5, 1:10, and 0:1, the predicted values of cumulative methane production from the mixture were 181.71, 354.61, 515.90, 389.26, and 248.40 mL/g VS, respectively, which were all larger than the experimental methane production values, and the differences that existed were 0.67%, 2.20%, 7.14%, 4.69%, and 8.50%. Likewise, the delayed response of the microorganisms to environmental changes was reflected by the delay period λ. For different mixing ratios, the delay times were 2.96, 3.69, 5.58, 4.69, and 6.83 days, indicating that the microorganisms in the inoculum needed 3–7 days to adapt to the environment in the system. All groups shortened the λ to different degrees and showed a gradual shortening of the λ as the proportion of pig manure increased; thus, the more pig manure that is added, the faster the gas production will be. The effective gas production time, T80, was the anaerobic digestion time when the cumulative gas production reached 80% of the total biogas production. The results showed that the T80 of the different mixing ratios was 14.13, 16.51, 19.11, 19.69, and 20.94 days. Among them, the mono-AD of rice straw showed a longer effective gas production time, indicating that more time was needed for complete fermentation, mainly due to the low microbial activity and organic matter conversion efficiency during the fermentation process. Therefore, the optimal mixing ratio was 1:5, and the cumulative methane production, λ, T80, and Rmax were more effective in this group than in other groups. Previous studies explored the effect of the mixing ratio on methane production during the AcoD of pig manure and rice straw [34,35]. For instance, Zhang et al. (2021) reported that the highest methane yield (188.79 mL/g VS) was obtained at a ratio of 1:1, which was higher than that of the mono-digestion of rice straw or swine manure by 178.77% and 18.94%, respectively.

The fitting curve between the cumulative methane production and the modified GM equation during the AcoD of the mixed materials with different substrate concentrations is shown in Figure 5b and Table 2. The results showed that the experimental values of cumulative methane production during fermentation for each treatment highly accorded with the predicted values of the modified GM equation when the initial substrate concentration was 6–12% (R^2^ > 0.995). However, when the initial substrate concentration was 14%, the experimental values of cumulative methane production in the reactor deviated significantly from the predicted values of the model, indicating that the equation was not suitable to describing the dynamics of methane production in fermentation systems with high substrate concentrations, which can be ascribed to the more complex relationship between the production and consumption of VFAs in fermentation systems with higher substrate concentrations. The predicted values of final methane production were 335.25, 412.65, 442.71, 476.35, and 265.11 mL/g VS at initial substrate concentrations of 6%, 8%, 10%, 12%, and 14%, respectively, with differences of 0.94%, 5.25%, 4.29%, 4.49%, and 6.06% from the experimentally detected values. Likewise, the maximum biogas production rate showed a trend of increasing and then decreasing when the substrate concentration increased. Moreover, the T80 values of each group were 15.43, 17.86, 17.52, 16.74, and 18.85 days, indicating that the greater the substrate concentration, the longer the delay period and the longer the fermentation cycle. This may be due to the fact that higher substrate concentrations and lower water content affect mass transfer and material transport, which are not conducive to mimicking the hydrolytic utilization of material. Previous studies reported that the primary kinetic hydrolysis constant of the studied material was 0.03–0.07/d in fermentation systems with substrate concentrations < 10%, while in fermentation systems with substrate concentrations > 15% of fibrous material, the constant was only 0.01–0.03/d, indicating that the degradation and conversion efficiency of the material was extremely low under conditions of high substrate concentrations [36]. Therefore, in the AcoD process, the initial substrate concentration of 12% was chosen to accelerate the reactor’s initiation, leading to a high degree of methanogenic efficiency.

The cumulative methane yield was fitted with the modified GM model for different inoculum conditions, and the fitting results and corresponding parameters are shown in Figure 5c and Table 2. The results showed that the cumulative methane yield of the fermentation process for each treatment was in high agreement with the predicted values of the modified GM model (R^2^ > 0.998). The predicted values of cumulative methane production were 260.85, 480.44, 587.52, 406.08, and 368.52 mL/g VS when the inoculum levels were 5%, 10%, 15%, 20%, and 25%, respectively, with differences of 4.78%, 8.14%, 6.09%, 3.58%, and 5.52%, respectively, from the experimental assay values. With the increase in the amount of inoculum, the maximum gas production rate showed a trend of increasing and then decreasing, with 17.33, 28.36, 36.85, 25.47, and 21.75 mL/g VS for each treatment group, while the delay times were 6.15, 5.67, 5.56, 3.64, and 3.33 days for each treatment group, and the T80 was 18.56, 19.64, 18.84, 18.64, and 18.81 days, respectively. The results showed that the delay in each treatment group shortened with the increasing amount of inoculum, with little effect on T80. The larger inoculum amount meant that more microorganisms entered the system, which led to the rapid growth and reproduction of the microorganisms in the system and the rapid decomposition of organic matter, thus reducing the delay period. In previous studies, the methane yield and kinetics of rice straw and pig manure anaerobic co-digestion under bio-pretreatment were explored [37]. The results showed that the maximum accumulative methane production of rice straw and pig manure reached 350.79 mL/g VS, which was 67.5% lower than in our study. However, the maximum gas production rate (45.36 mL/g VS) and delay time (1.79 days) of rice straw and pig manure anaerobic co-digestion after bio-pretreatment were higher and lower than those of our study, respectively. This may be because the biological pretreatment made the substrate more readily available for degradation and conversion, thus shortening the delay period and increasing the maximum gas production rate [38]. These results provide ideas for the subsequent combination of parameter optimization and pretreatment measures to optimize the system performance of anaerobic co-digestion.

### 3.5. Characteristic of Cooperative Index of Co-Anaerobic Digestion of Pig Manure and Rice Straw

**Figure 6:**
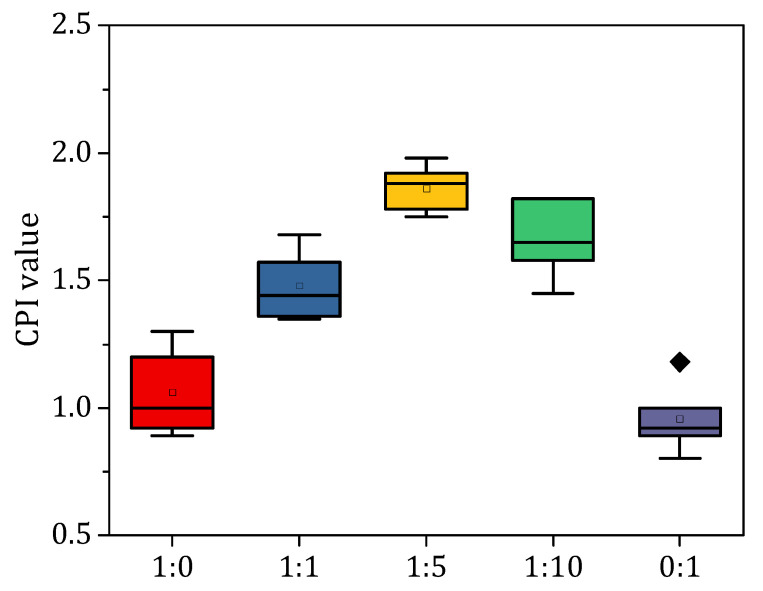
Results of cooperative index of anaerobic co-digestion of pig manure and rice straw under different mixing ratios.

In this study, the cooperative index (CPI) was used to indicate the synergistic effect of pig manure and rice straw co-digestion (Figure 6). Generally, CPI > 1 was considered a synergistic effect and CPI < 1 was considered an inhibitory effect. The results showed that the CPI value was greater than 1 in all AcoD processes except the mono-anaerobic digestion process, and the CPI values ranged from 1.44~1.87. This result indicated that the co-digestion of the mixture of the two materials promoted the methanogenic process. When the ratios of pig manure and rice straw were 1:1, 1:5, and 1:10, the CPI values were 1.44, 1.87, and 1.62, respectively, which were 44.21%, 87.15%, and 62.47% higher than the calculated value of cumulative methanogenesis. With the increase in the straw proportion, the synergistic effect showed a trend of increasing and then decreasing, in which the highest synergistic effect value was found at the mixing ratio was 1:5, which significantly increased the methane production of the anaerobic digestion system by 42.94% and 24.68% compared with the other mixing ratios. The above results show that AcoD is not only a simple substrate superposition but can also play a remarkable role with respect to synergistic promotion, and that the appropriate ratio of rice straw can significantly enhance the synergistic effect. The co-digestion of pig manure and rice straw improved methane production, and the optimal ratio of 1:5 under the experimental conditions resulted in a more balanced nutrient composition of the fermented substrate, enriched the diversity of microorganisms, and significantly enhanced the synergistic effect.

## 4. Conclusions

In view of the prevalence of nutrient imbalance and low methane production efficiency in the single anaerobic digestion of pig manure and rice straw, this study investigated the process parameters of the anaerobic co-digestion of pig manure and rice straw. The results showed that the optimal process parameters were as follows: a 1:5 mixing ratio, 12% TS content, and 15% activated sludge inoculation determined via methane production levels, pH values, and VFA determination. Likewise, a significant synergistic effect was found in the process of the co-digestion of pig manure and rice straw through the determination of the CPI. Not only can this finding advance our understanding of the process of the co-digestion of pig manure and rice straw, but it can also support research toward improving anaerobic co-digestion performance by optimal process parameters.

## Figures and Tables

**Table 1:** The characteristics of pig manure, rice straw, and inoculum.

Parameters	Pig Manure	Rice Straw	Inoculum
TS (%)	31.93 ± 1.03	94.79 ± 1.12	13.57 ± 0.13
VS (%)	25.03 ± 4.34	81.80 ± 0.97	5.33 ± 0.15
VS/TS (%)	78.37 ± 1.05	86.29 ± 0.24	39.26 ± 1.73
pH	7.86 ± 0.11	/	7.58 ± 0.05
TC (%/TS)	37.91 ± 0.43	47.25 ± 0.55	21.23 ± 0.84
TN (%/TS)	4.63 ± 0.56	1.18 ± 0.76	4.15 ± 0.47
C/N	8.18 ± 0.19	40.04 ± 0.15	5.12 ± 0.26
Cellulose (%/TS)	/	36.15 ± 1.27	/
Hemicellulose (%/TS)	/	24.13 ± 1.62	/
Lignin (%/TS)	/	21.38 ± 0.93	/
Ash content (%/TS)	9.56 ± 1.32	10.12 ± 1.18	/

TS: Total solids; VS: Volatile solids; TN: Total nitrogen; TC: Total carbon.

**Table 2:** The kinetic parameters estimated at the different conditions.

	P (mL/g VS)	Rmax (mL/d·g VS)	T_80_	λ (d)	R^2^
Mixing ratio					
1:0	181.71	14.75	14.13	2.96	0.998
1:1	354.61	23.55	16.51	3.69	0.999
1:5	515.90	30.46	19.11	5.58	0.999
1:10	389.26	27.29	19.69	4.69	0.997
0:1	248.40	13.75	20.94	6.83	0.998
TS content					
6%	335.25	28.45	15.43	3.88	0.999
8%	412.65	28.93	17.86	4.14	0.998
10%	442.71	31.04	17.52	4.51	0.997
12%	476.35	31.64	16.74	5.43	0.997
14%	265.11	14.67	18.85	5.86	0.999
Inoculum amounts					
5%	260.85	17.33	18.56	6.15	0.998
10%	480.44	28.36	19.64	5.67	0.999
15%	587.52	36.85	18.84	5.56	0.999
20%	406.08	25.47	18.64	3.64	0.996
25%	368.52	21.75	18.81	3.33	0.998

P is the final accumulative methane production (mL g^−1^ VS), Rmax is the maximum methane production rate (mL g^−1^ VS-d^−1^), T80 refers to the effective gas production, R^2^ is the fit index, and λ is the lag phase time (days).

## Data Availability

Data are available from the authors.
